# A Comparison of Energy-Efficient Seizure Detectors for Implantable Neurostimulation Devices

**DOI:** 10.3389/fneur.2021.703797

**Published:** 2022-03-04

**Authors:** Farrokh Manzouri, Marc Zöllin, Simon Schillinger, Matthias Dümpelmann, Ralf Mikut, Peter Woias, Laura Maria Comella, Andreas Schulze-Bonhage

**Affiliations:** ^1^Epilepsy Center, Department of Neurosurgery, Medical Center — University of Freiburg, Faculty of Medicine, University of Freiburg, Freiburg, Germany; ^2^Laboratory for Design of Microsystems, Department of Microsystems Engineering — IMTEK, University of Freiburg, Freiburg, Germany; ^3^Institute for Automation and Applied Informatics, Karlsruhe Institute of Technology, Eggenstein-Leopoldshafen, Germany; ^4^Faculty of Medicine, Center for Basics in NeuroModulation, University of Freiburg, Freiburg, Germany

**Keywords:** seizure detection, responsive neurostimulation, low-power hardware implementation, random forest, recurrent neural network, convolutional neural network

## Abstract

**Introduction:**

About 30% of epilepsy patients are resistant to treatment with antiepileptic drugs, and only a minority of these are surgical candidates. A recent therapeutic approach is the application of electrical stimulation in the early phases of a seizure to interrupt its spread across the brain. To accomplish this, energy-efficient seizure detectors are required that are able to detect a seizure in its early stages.

**Methods:**

Three patient-specific, energy-efficient seizure detectors are proposed in this study: (i) random forest (RF); (ii) long short-term memory (LSTM) recurrent neural network (RNN); and (iii) convolutional neural network (CNN). Performance evaluation was based on EEG data (*n* = 40 patients) derived from a selected set of surface EEG electrodes, which mimic the electrode layout of an implantable neurostimulation system. As for the RF input, 16 features in the time- and frequency-domains were selected. Raw EEG data were used for both CNN and RNN. Energy consumption was estimated by a platform-independent model based on the number of arithmetic operations (AOs) and memory accesses (MAs). To validate the estimated energy consumption, the RNN classifier was implemented on an ultra-low-power microcontroller.

**Results:**

The RNN seizure detector achieved a slightly better level of performance, with a median area under the precision-recall curve score of 0.49, compared to 0.47 for CNN and 0.46 for RF. In terms of energy consumption, RF was the most efficient algorithm, with a total of 67k AOs and 67k MAs per classification. This was followed by CNN (488k AOs and 963k MAs) and RNN (772k AOs and 978k MAs), whereby MAs contributed more to total energy consumption. Measurements derived from the hardware implementation of the RNN algorithm demonstrated a significant correlation between estimations and actual measurements.

**Discussion:**

All three proposed seizure detection algorithms were shown to be suitable for application in implantable devices. The applied methodology for a platform-independent energy estimation was proven to be accurate by way of hardware implementation of the RNN algorithm. These findings show that seizure detection can be achieved using just a few channels with limited spatial distribution. The methodology proposed in this study can therefore be applied when designing new models for responsive neurostimulation.

## Introduction

### Problem Definition

Epilepsy is a brain disorder characterized by recurrent epileptic seizures (1) and is one of the most common neurological diseases, affecting nearly 70 million people worldwide (2). Epileptic seizures are defined as episodes of excessive or abnormal synchronous neuronal activity in the brain, and can be accompanied by clinical neurological symptoms such as abnormal movements, abnormal sensory phenomena, loss of consciousness, or alterations in consciousness. Epilepsy is therefore associated with considerable neurological morbidity. Epileptic seizures can vary in form, not only between different patients but also within a single patient.

Despite advances in the development of medication, about 30% of epilepsy patients are resistant to a treatment with antiepileptic medications (3). Nevertheless, only 7–8% of these patients are surgical candidates (4).

In the case of focal seizures, surgical resection of the brain region(s) generating the seizures may be used to prevent further seizures. Nonetheless, because not all of these patients have a unifocal seizure onset zone (SOZ) and the epileptogenic brain tissue cannot always be resected without significant functional loss, more innovative therapeutic approaches are urgently required.

A recently proposed treatment approach for these patients is electrical stimulation of the epileptic focus during the early phases of a seizure in order to interrupt its spread across the brain (2). This can be accomplished using a closed-loop neurostimulation implant, which records electrical brain activity via a set of electrodes and continuously monitors electroencephalography (EEG) activity applying a seizure detection algorithm. It then triggers electrical stimulation at the SOZ via the same electrodes in the advent of an emerging seizure. This approach requires the early detection of seizures with high accuracy based on EEG. Furthermore, the selected seizure detection algorithm should be computationally efficient to have a low energy consumption for long-term application in an implantable, battery-powered device.

Currently, the only FDA-approved implantable device for clinical applications that applies this principle is the so-called “RNS device” (Neuropace Inc., USA). This device provides electrical stimulation via a generator fixed to the skull, with electrodes within or directly above the cortical region of the epileptic focus (1). Whereas, this device has proven to be efficacious both under short-term and long-term applications (3, 5, 6), its implantation is complex and the risk of infection at the implantation site is high, due to the intracranial placement of the electrodes (4).

In addition, the RNS device suffers from a high number of false detections. As false detection rates (FDRs) of the device are not reported explicitly, they can only be derived from the length of reported stimulation times. According to Heck et al. (3) a mean stimulation period of 5.9 min was applied. With a pulse burst duration of 100 ms, this corresponds to 3,540 stimulations per day and a minimum of 354 detections per day and 10,620 detections per month. At a baseline seizure frequency of 8.7 per month, this corresponds to >1,220 false detections per crisis event. This high number of interventions leads to the question of how much efficacy is related to closed-loop suppression of seizure-related ictal activity, in contrast to long-term depression of seizure probability by neuromodulation. In addition, improved seizure detection algorithms with higher specificity of interventions can lead to a longer neurostimulator battery life, due to the reduced number of stimulations; accordingly, it may lead to fewer side effects of the stimulation.

### Previous Studies

The development of seizure detection algorithms based on EEG data began several decades ago (7). The initial objective was to reduce the workload of reviewing continuous long-term recordings in epilepsy monitoring units and presenting the neurologist with intervals of only the highest clinical relevance. In addition, more recent studies have addressed the development of seizure detection algorithms for responsive stimulation to prevent the onset or spread of seizure activity in the early phase of a seizure (8). Such application scenarios require a reliable seizure detector at a reasonable computational load. Furthermore, performing an intervention exactly at the onset of a seizure, requires early detection, which is considered in more recent approaches (9–11). Because of the high variation in EEG patterns that characterize a seizure (12), the large variability in background EEG activity among patients, and the intra-individual fluctuations in EEG activity, the problem of seizure detection remains an active research topic (13–15). While several publications have proposed seizure detection algorithms for either offline or online applications, only a limited number of studies have addressed the limitations of seizure detection for closed-loop applications. One particular limitation is the use of only a few electrodes for seizure detection, which is driven by properties of the neurostimulator. Seizure detection using a low number of electrodes has recently achieved increased interest, as this concept enables mobile seizure monitoring (16–18). In contrast, outpatient monitoring with a complete electrode setup is seen as stigmatizing—as in any application that exposes the patient to the public—and is therefore not feasible in practice. A recent study by Vandecasteele et al. (19) described a seizure detection algorithm based on a support vector machine classifier using behind-the-ear EEG data derived from only four electrodes. However, although the proposed model had a relatively low FDR, it also had low sensitivity (19). In another recent study, Dan et al. (20) proposed a method for detecting electrographic patterns during absence seizures (short, non-motor generalized onset seizures); this was based on a linear multichannel filter which was precomputed with the spatiotemporal signature of the seizure and the peak interference statistics that could run on a microcontroller. Nevertheless, this particular method requires 20 recording channels. Therefore, the need to develop a seizure detection algorithm that can detect seizures in early stages not only with high sensitivity and specificity, but also with low computational power and a limited number of electrodes has not yet been fulfilled.

Regarding energy estimation of the seizure detection algorithms, several methods have been introduced. García-Martín et al. (21) reviewed the current approaches used to estimate energy consumption in machine learning. They suggested dividing the common methods of energy estimation into three different categories, namely at the software-application level, the software-instruction level, and the hardware level (21). The software categories describe the energy related to the algorithm features, and are based either directly on the application level, or on the underlying instructions. Here, a model links the instructions to the energy demand. The hardware level relates the energy caused by the application to the components in the hardware setup. Among the methods, the instruction level in the software category is most relevant to the scope of this study. At the instruction level in particular, the energy consumption is evaluated on the basis of program-specific instructions. This approach was followed by Rouhani et al. (22), in which the power consumption of a deep neural network (DNN) was estimated using the number of multiply-accumulate (MAC) operations. Likewise, Taghavi et al. (23) estimated the hardware requirements of DNNs and decision tree ensembles in terms of MAC operations and parameters required for the classification model. They compared several studies using energy efficiency, hardware complexity and classification performance as parameters (23). Yang et al. (24) applied this approach to estimate the energy consumption for a CNN algorithm. They constructed the total energy consumption by first extending the model based on MAC operations and model parameters with the number of memory accesses (MAs). Next, they weighted these parameters with measured MAC operations and MA energies to calculate the total energy dissipation (24).

### Own Approach

**Figure 1:**
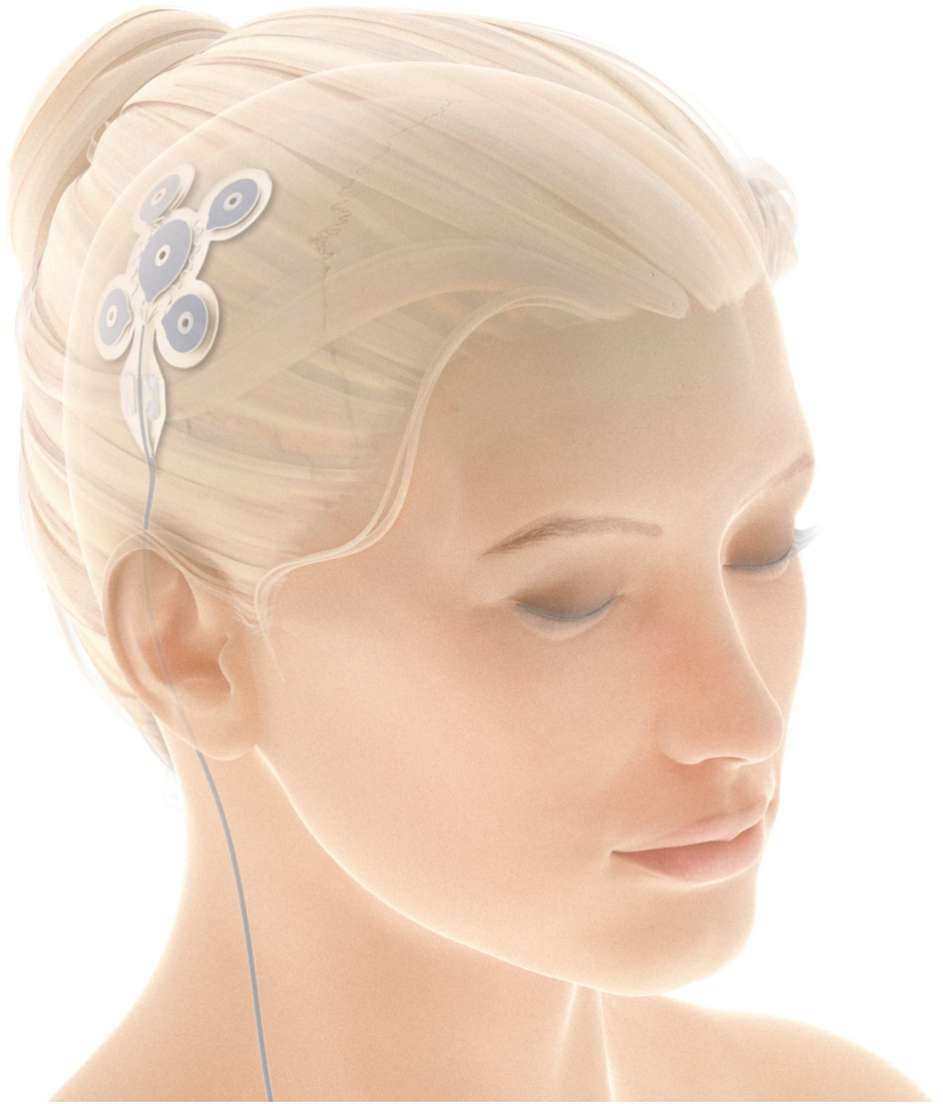
A minimally invasive electrode setup as a part of an implantable system for focal epilepsy (Copyright © Precisis AG, Heidelberg, Germany).

A minimally invasive, implantable neurostimulation approach was recently developed using subgaleally placed stimulation electrodes (EASEE System, Precisis AG, Heidelberg). The subcutaneous system uses electrodes that are placed outside the cranium over the SOZ, and connected to a pulse generator on the trunk (Figure 1). For the integration of a seizure detection algorithm into this kind of fully implantable intervention device, several limitations must first be considered. The seizure detection algorithm must perform well despite a low number of electrodes, limited spatial coverage, and low computational power. To address this issue, three patient-specific seizure detection algorithms that only apply four channels each were developed and their performance was evaluated: random forest (RF), convolutional neural network (CNN), and long short-term memory (LSTM) recurrent neuronal network (RNN). In addition, to evaluate their suitability for application in an implantable, responsive neurostimulator, their respective computational load and power consumption were estimated. As in this study, the classification rate is 1 Hz; the estimated energy demand in μJ is equal to the power consumption in μW. To estimate the required computational load, an instruction-level model based on application-specific instructions and MAs was developed and validated.

The quality of subcutaneous recordings was compared to standard surface EEG recordings by Duun-Henriksen et al. (25). They showed that some aspects of the subcutaneous recordings might be similar, or even superior, to surface EEG recordings (25). Likewise, Weisdorf et al. (26) reported a high similarity between EEG data from subcutaneous and proximate scalp electrodes in patients with temporal lobe epilepsy. Accordingly, the seizure detection algorithms developed in the current study were tested on surface EEG data from electrodes configured to resemble the subgaleal electrode placement.

## Materials and Methods

**Figure 2:**
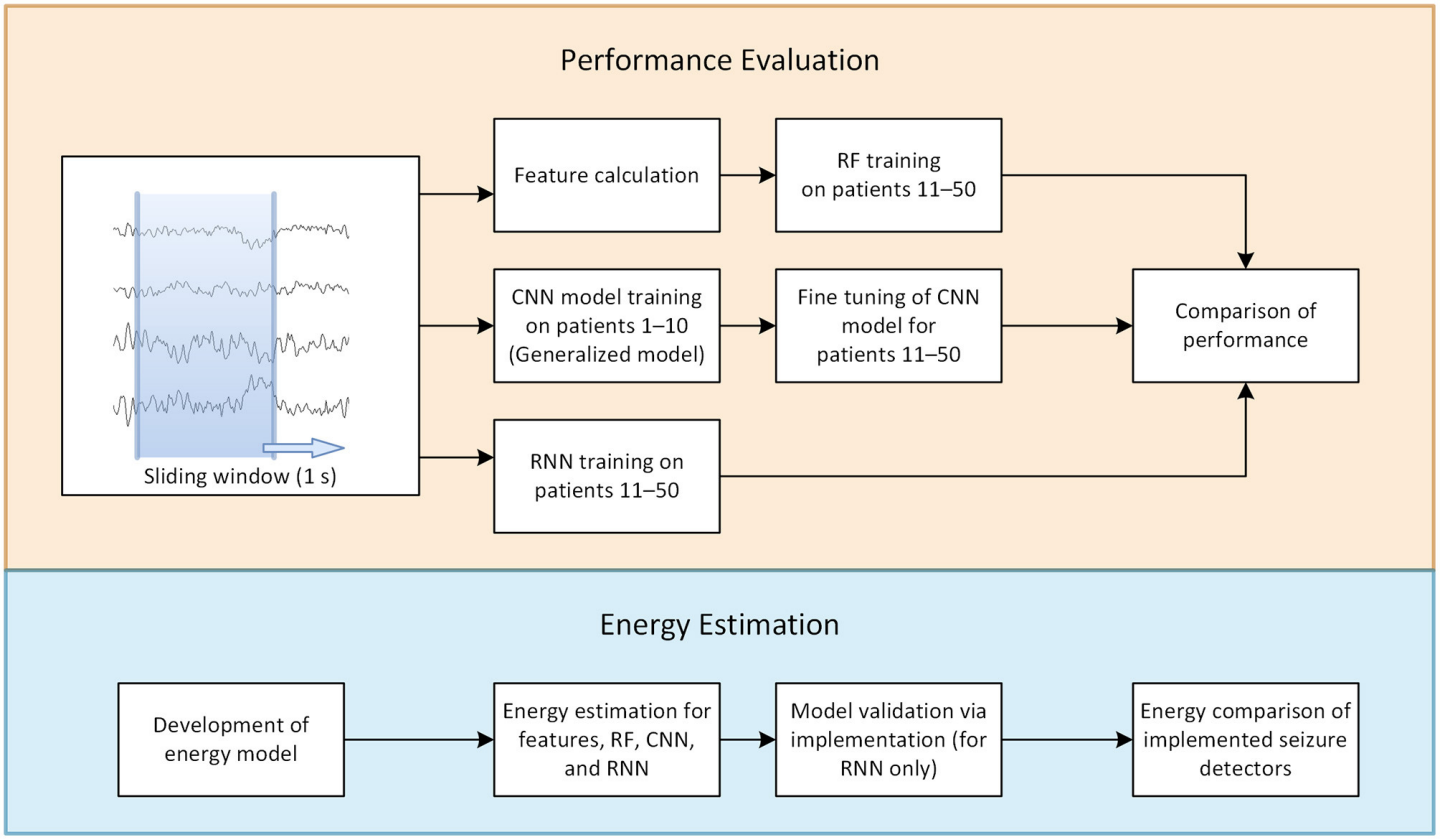
Schematic outline of the study design.

A schematic outline of the study design is presented in Figure 2. In the following sections, the methodology used for evaluating performance and estimating power consumption is described. A more detailed description of the energy estimation method is provided in the Supplementary Material section.

### Dataset

The dataset consists of surface EEG recordings from 50 patients (23 female; age range, 14–66 years; mean age, 32.5 years) with focal-onset seizures at the Epilepsy Center Freiburg, Germany; the total number of seizures was 357. Figure 2 demonstrates how the dataset was split for training the different classifiers, i.e., patients 1–10 were selected for pre-training (CNN only), while the remaining 40 patients (11–50) were selected for training and evaluation (all 3 methods). Due to considerable imbalance between the ictal and interictal classes for each seizure, an hour-long time window around each seizure was selected for evaluation and the remaining data were then excluded from analysis. Patients were selected based on long-term video recordings that included at least five seizures, with electrodes positioned over the SOZ according to the 10–10 electrode layout (27). Electrode selection was performed to represent device layout. Expert epileptologists defined the SOZ by visual exploration of the seizure onset electrodes recordings, taking into account the inter-electrode distances. The study was approved by the local Ethics Review Board. Informed consent of the patient covered the in-house use of EEG recordings. EEG data were recorded at a 256 Hz sampling rate on a 256-channel DC amplifier with 24-bit resolution (Compumedics, Abbotsford, Australia). For anti-aliasing, a low-pass filter with a cut-off frequency of 100 Hz was applied. Five electrodes covering the SOZ were selected for seizure detection in each patient.

### Data Preprocessing

A small number of preprocessing steps were applied to reduce the effects of noise and artifacts on the performance of the seizure detection algorithms. First, invalid segments of the EEG signals, including segments where the signal was not recorded due to electrode deficiency or during the electrode impedance measurement, were excluded from analysis. Next, to improve the signal quality, a 10th order Chebyshev Type-II IIR (infinite impulse response) bandpass filter with a stopband attenuation of 40 dB and respective stopband frequencies of *f*_1_ = 0.1 *Hz*, and *f*_2_ = 48 *Hz* was applied. The settings were chosen to filter power line noise and reduce the influence of broad-band muscle activity, spanning up to 200 Hz (28). To remove high-amplitude sharp artifactual transients, another artifact rejection step was added in which periods of *T*_*p*_ = 1 *s* that contain amplitudes higher than 1 mV were removed. This threshold was set in such a way that allowed amplitudes in the range of 100 μV [typical of EEG signals in healthy subjects (29)], as well as interictal spikes up to ~140 μV (30) to remain in the data. Moreover, the selected threshold value allows a good trade-off between excluding artifacts vs. retaining the scarcely represented ictal patterns in the data, so as to avoid further imbalance in the dataset. Finally, to account for the locality of the target electrode configuration, the EEG channels were re-referenced by subtracting the recordings of the centrally positioned electrode from those of each of the peripheral electrodes.

### Seizure Detection

Seizure detection can be modeled as a time-series classification in which ictal phases (seizures) and interictal phases (non-seizures) are classified. In this section, the three proposed supervised-learning algorithms for seizure detection are detailed.

#### Random Forest (RF)

RF is an ensemble-learning method for classification or regression that operates by constructing a group of decision trees in which each tree is grown using binary decisions (each parent node is split into two children) (31). This method combines the “bagging” technique with the random selection of features. The randomness of each tree is accomplished in two ways: first, by random selection of a subset of approximately two thirds of the data for training the tree, and second, by feature selection of the nodes of each tree, which is done using a randomly selected subgroup of features. The remaining one third of the training data is used for out-of-bag error evaluation and performance calibration of the tree. In this study, the number of binary decision trees was set to 100. Entropy was selected as the branching index for growing each decision tree. The best feature (splitter) from the eligible, randomly selected subset of features, which has the highest importance, is used to split the node. In line with the Liaw and Wiener (32) results, the optimal number of randomly selected features at each tree node is sqrt(N), where N is the number of features. In this study, 16 time- and frequency-domain features were calculated from the EEG data as input. The time-domain features were mean, maximum, mean absolute deviation (MAD), variance, skewness, kurtosis, line length and entropy. Frequency-domain features included values for the maximum, mean, and variance of the power spectrum, power in the theta (4–8 Hz), beta (13–30 Hz), and gamma band (30–45 Hz), spectral entropy, and epileptogenicity index, the latter of which is defined as the ratio of power in the higher frequency bands (beta + gamma) vs. the lower frequency bands (theta + alpha) (33). As a result, four features were randomly selected at each decision tree node. To maintain a limited tree size and to confine the required memory for hardware implementation, the maximum depth of the decision tree was limited to 10. Bootstrap samples were used while building the decision trees. The sample weights for each class were adjusted to be inversely proportional to the class frequencies in the training data. A non-overlapping time window of 1 s was selected for seizure detection. For implementation of the RF, the freely available and open source Scikit-learn machine learning library was used (34).

#### Convolutional Neural Network (CNN)

CNN is a class of DNNs that were inspired by biological processes (35) and are commonly applied for pattern recognition (36). A CNN consists of an input layer, multiple hidden layers, and an output layer. The hidden layers consist of convolutional layers, pooling layers, and potentially fully connected layers. In the convolutional layers, the input is convolved with filters to detect patterns, and the results are conveyed to the activation function which is usually a rectified linear unit (ReLU). In addition, CNNs may include local or global pooling layers that reduce the dimensions of the data. The pooling layer helps to control overfitting by making the pattern representation almost invariant to minor translations of the input. This is accomplished by striding a window over the output of the activation function and pooling the average or maximum value. Subsequently, the results matrix is flattened and fed to the fully connected layers to drive the classification decision.

To create the inputs for the convolutional network, sliding windows of 1 s over the EEG data were processed. First, the data was rescaled by dividing it by the standard deviation of the training data. Next, the data was normalized using an estimation of the hyperbolic tangent function, as shown in the following equation:


$$\begin{array}{l} \hat{x}\left(t\right)=\tanh\left(0.2\cdot x\left(t\right)\right) \end{array}$$

To facilitate hardware implementation of the hyperbolic tangent function and avoid the need for a lookup table, a linear approximation of the hyperbolic tangent was applied for classification:


$$\begin{array}{l} \text{lintanh(x)}:=\{\begin{array}{ll} x/1.2\; & -1.2\leq x\leq 1.2\\ 1\; & x>1.2\\ -1\; & x<-1.2\\ \; \end{array} \end{array}$$

The architecture of the proposed CNN in this study is shown in Table 1. Four channels of simultaneously recorded raw EEG data with a duration of 1 s (256 data points) were selected as the input. In the first convolutional layer, a kernel size that extends over time and all four EEG channels was used to facilitate efficient learning of the spatiotemporal patterns. No padding was applied in the convolutional layers. In all hidden layers, batch normalization was applied after the convolutions were performed (37). The ReLu was selected as the activation function. Dropout regularization was used during training to reduce overfitting and generalization error (38). In the last two layers, two fully connected layers were employed.

**Table 1:** Architecture of the proposed CNN.

**Layer**	**Operation**	**Output**	**Parameters #**
	Input (C ×256)	C ×256 ×1	–
1	15 × Conv2D (C ×25)	1 ×232 ×15	1,515
	Batch Normalization	1 ×232 ×15	60
2	MaxPool2D (1 ×4)	1 ×58 ×15	0
	Dropout (0.2)	1 ×58 ×15	0
3	15 × Conv2D (1 ×11)	1 ×48 ×15	2,490
	Batch Normalization	1 ×48 ×15	60
4	MaxPool2D (1 ×4)	1 ×12 ×15	0
	Dropout (0.2)	1 ×12 ×15	0
5	10 × Conv2D (1 ×5)	1 ×8 ×10	760
	Batch Normalization	1 ×8 ×10	40
	Dropout (0.2)	1 ×8 ×10	0
6	Dense (8)	8	648
7	Dense (4)	4	36
8	Sigmoid	1	5

*C, Number of channels*.

Due to the limited amount of available data for each patient, the transfer learning method was applied for training the model. This is generally done by applying the gained knowledge from a learning problem to improve learning on a related problem. Accordingly, the model was first pre-trained with data from 10 patients. For each of the remaining 40 patients, the model was subsequently fine-tuned using patient-specific data. Because the classes (ictal vs. interictal) were imbalanced, the class indices were weighted to balance the weighting of the loss function during the training phase (39). Each model was trained for 500 epochs with a batch size of 512. For weight optimization, an Adam solver (40) with a learning rate of 10^−^3 was used. Binary cross-entropy was selected as the loss function.

#### Recurrent Neural Network (RNN)

Recurrent Neural Networks (RNNs) are a class of neural networks with recurrent connections that allow the network to store information over time. RNNs started to gain attention with the introduction of LSTM cells, which significantly improves their performance. The LSTM cell was first introduced by Hochreiter and Schmidhuber (41) to overcome the problem of vanishing gradients in RNNs. With its internal state, the LSTM cell is capable of storing information over time, depending on its input and output values. The flow of information is controlled by the so-called gates that can learn which data in a sequence is important to remember or disregard. Gates can be seen as controlling valves with the ability to let the information pass through by multiplying it by the gate values. These features allow LSTM cells to be used in time-series problems. Combined with other neural network layers, they form an LSTM neural network. Such networks are used across many different applications and are considered especially useful in sequence prediction or sequence classification problems (42).

The proposed RNN architecture is based on the work of Hussein et al. (43) with a few modifications to reduce the complexity of the model. Similar to CNN, four channels of simultaneously recorded raw EEG data with the duration of 1 s (256 data points) were selected as the input. As shown in Table 2, the network consists of an LSTM layer containing 20 cells with an input size of 256. Similar to the CNN, a dropout layer is used for regularization. This is followed by a time-distributed dense layer consisting of 20 dense cells with a linear activation function that is computed at every time step. Subsequently, the global average pooling layer averages the dense cell outputs over time. Finally, as this is a binary classification task, an output dense layer with a sigmoid activation function is used that simplifies the calculation.

**Table 2:** Architecture of the proposed RNN.

**Layer**	**Operation**	**Output**	**Parameters #**
	Input (C ×256)	C ×256 ×1	–
1	LSTM (20)	256 ×20	2,000
	Dropout (0.1)	256 x 20	0
2	Time-Distributed Dense (20)	256 x 20	420
3	Global Average Pooling 1D	20	0
4	Dense (1)	1	21

*C, Number of channels*.

The classifier performance was investigated with both raw data and its time-derivative for each individual channel. The latter was calculated through the difference of subsequent values for all electrodes and all time steps as:


$$\begin{array}{l} \Delta x_{c_{k}}=x_{c_{k}}-x_{c_{k-1}} \end{array}$$

where *c* corresponds to the electrode number and *k*∈{1, …, *K*} is the sample index with *K* samples in the corresponding seizure. Next, the training data were prepared by striding a moving window over the sample axis of the input data. For training, a step size of *stride*_*inter*−*ictal*_ = 16 was chosen for the interictal class, and a *stride*_*ictal*_ = 1 was used for the ictal class; this was done both to account for the imbalance and to increase the ictal sample size. For validation, a single seizure that was excluded from the training data was used with the same stride settings as those used for training. The test set was created with a moving window step size of *stride* = 256. Furthermore, early stopping (44) was applied as another regularization technique to prevent overfitting and reduce training time. The patience parameter was set to 10 epochs. It defines the maximum number of epochs than may occur until an improvement in the validation dataset is observed, before stopping the training process. Validation loss was used as the performance metric to decide whether early stopping was necessary. The samples were presented with a mini-batch size of 256. The binary cross entropy was chosen as the loss function and the Adam optimizer chosen to tune the learning rate. Finally, to further improve the classifier performance, the output probabilities were median filtered with a moving median size of three.

#### Performance Evaluation

For performance evaluation of the seizure detection algorithms, the “leave-one-out” method was used for cross-validation, whereby in every iteration one seizure was selected for testing and all remaining data were used for training. The EEG data from ten patients were used to pre-train the CNN model. Performance evaluation was conducted across the remaining 40 patients (total number of seizures = 286) for each of three proposed classifiers. Evaluation metrics were calculated for each patient separately and then averaged across all the patients. Metrics for the performance evaluation of seizure detection algorithms are either dependent on a threshold-level set for the seizure probability, or are threshold-free. Accepted threshold-dependent metrics for performance evaluation of the seizure detectors are sensitivity, FDR, and average detection delay. For calculation of the sensitivity, a seizure was counted as correctly detected when it was detected at least once during the ictal phase. Regarding the FDR, false detections within a 5 s window were counted only once, since they generally refer to the same detected pattern. Nevertheless, there is an inevitable trade-off between these threshold-dependent evaluation metrics. For example, a higher sensitivity and lower detection delay can be attained if a higher FDR is accepted. While the use of these metrics facilitates the estimation of seizure detector performance in different application scenarios, the use of threshold-free metrics simplifies the comparison of the classifier performance by combining threshold-dependent metrics into one threshold-free metric. To yield a threshold-free comparison of the classifiers, the area under the curve (AUC) of the receiver operating characteristic (ROC) and precision-recall (PR) curves were selected as performance metrics. For calculating threshold-free metrics, the true and false detections were counted for each time window (1 s), which is different from how the threshold-dependent metrics were calculated. Considering that detection delay is not projected in the AUC, a modified version of the selected metrics was used as additional metrics, denoted as “early seizure AUC.” In this case, only those seizures detected within the first 10 s of seizure onset (as determined by the epileptologist) were deemed successfully detected.

### Energy Estimation

The energy estimation of the proposed seizure detection algorithms in this study is based on the number of arithmetic, memory read, and store operations. The total energy required was estimated via the energy costs per operation (24). The method in Yang et al. (24) was modified to use the energy estimation per operation based on the findings reported in Horowitz (45), instead of measuring the energy for specific hardware. Additional measurements were performed, and assumptions were made for operations not included in their proposed method. Table 3 shows an overview of the operations and corresponding energy consumption relevant for the developed model. Horowitz (45) defines the rough energy cost in a 45 nm process technology for the fundamental operations and MAs. In addition, Horowitz included the energy cost of the microprocessor overhead, which deliberately was not taken into account in the current study for the purpose of obtaining a hardware-independent measure. Nevertheless, the proposed model can be adapted to specific hardware if required. In addition, it enables the identification of operations that have a higher impact on energy consumption. Hence, it helps in implementing efficient signal processing algorithms, or aids in selecting the most suitable microprocessor.

**Table 3:** Energies assumed for the estimation of the power consumption.

**Parameter**	**Energy**
*E*_*m*_	5 *pJ*
*E*_*int*32−*add*_	0.1 *pJ*
*E*_*int*32−*mult*_	3.1 *pJ*
*E*_*float*32−*add*_	0.9 *pJ*
*E*_*float*32−*mult*_	3.7 *pJ*
${E_{compare}}^{\text{a}}$	0.9 *pJ*
${E_{float32-divide}}^{\text{b}}$	26.3 *pJ*
${E_{float32-1-cycle}}^{\text{c}}$	3.7 *pJ*

*E_m_ is half the cache energy estimated by the energy cost table for 45 nm at 0.9 V from Horowitz (45) for an 8 kB 64-bit wide cache access, as only 32-bit loads are considered in this study. E_float32−add_, E_float32−mult_, E_int32−add_, E_int32−mult_ values are also from Horowitz (45).*

*^a^As a compare operation can be performed as a subtraction which is basically an addition, the same energy is assumed for the compare operation. ^b^A benchmark on the Ambiq Apollo 3 Blue with ARM Cortex M4F processor measured by executing three different measurements with a loop containing float multiply, divide and both instructions to cancel out loop and other overhead, showed a factor of 7.1 of float division energy consumption compared to float multiply energy consumption. ^c^Other floating-point operations that take one clock cycle are considered to consume as much as a multiply operation*.

The total energy is calculated as $E_{tot}=\left(N_{load}+N_{store}\right)\cdot E_{m}+\underset{x}{\sum }N_{x}\cdot E_{x}$, where *N*_*load*_ is the number of load operations, *N*_*store*_ the number of store operations, *E*_*m*_ the MA energy, *N*_*x*_ and *E*_*x*_ the number of operation x and the corresponding energy defined in Table 3.

The following assumptions were additionally made: (1) the energy required for a MAC operation *E*_*MAC*_ is defined as *E*_*MAC*_ = *E*_*add*_+*E*_*mult*_. (2) The energy overhead of the square root function, which is not included in Horowitz (45), corresponds to that of a division operation. This assumption is based on the specification of the ARM Cortex-M4F floating-point unit, which states that the two operations have the same number of execution cycles.

#### Random Forest Energy Estimation

For energy estimation of the RF classifier, the number of required arithmetic and memory operations for the 16 time- and frequency-domain features were first calculated. A 32-Bit floating-point arithmetic was chosen to obtain an energy estimation value comparable to that of the RNN and CNN. Divisions by a constant are considered as multiplications, as their energy consumption is lower compared to a division. Each feature is considered to be computed over the sample or time axis in the input data array $v_{in}\in {\mathbb{R}}^{N_{w}\times C}$, for *N*_*w*_ = *F_s_*·*T*_*w*_, where *F*_*s*_ is the sampling frequency and *T*_*w*_ is the length of the window required for an observation. In this section, individual energy considerations are made for a single input channel. Consequently, the total energy is calculated by multiplying the required energy for a single channel with the number of channels C, assuming that all features are calculated for each channel.

##### Time-Domain Features

To improve computational efficiency and avoid redundant calculations, zero-mean values of the signal and their square values were calculated only once, and then shared along a set of features. The required number of arithmetic operations (AOs) and MAs were estimated based on the number and type of mathematical operations needed to calculate the features. Table 4 shows the equations that were considered for the calculation of the time-domain features, with x being the vector containing the input samples.

**Table 4:** Assumed equations for the Time-domain features.

**Time-Domain feature**	**Equation**
Maximum	max − val = *max*(*x*)
*Mean*	$\bar{x}=\frac{1}{N_{w}}\cdot \overset{N_{w}}{\underset{i=1}{\sum }}x_{i}$
*MAD*	$MAD=\frac{1}{N_{w}}\overset{N_{w}}{\underset{i=1}{\sum }}|x_{i}-\bar{x}|$
Variance	$var=\frac{1}{N_{w}}\overset{N_{w}}{\underset{i=1}{\sum }}{\left(x_{i}-\bar{x}\right)}^{2}$
Skewness	$g_{m}\;=\frac{1}{{N_{w}\cdot s}^{3}}\overset{N_{w}}{\underset{i=1}{\sum }}{\left(x_{i}-\bar{x}\right)}^{3}$
Kurtosis	$w\;=\frac{1}{{N_{w}\cdot s}^{4}}\overset{N_{w}}{\underset{i=1}{\sum }}{\left(x_{i}-\bar{x}\right)}^{4}$
Line Length	$LL=\overset{N_{w}}{\underset{k=2}{\sum }}|x_{k}-x_{k-1}|$
Entropy	$H=-\sum_{i}p_{i}\cdot log_{2}(p_{i})$

##### Frequency-Domain Features

The calculation of the frequency-domain features is based on the power spectrum *P*∈ℝ^*L*^, where L is the number of frequency bins. It is obtained using the squared values of the Discrete Fourier Transform (DFT) of the windowed raw time-domain signal *x*(*t*). To minimize spectral leakage, a Hanning-window was applied before performing the DFT. The equations of the frequency-domain features are summarized in Table 5.

**Table 5:** Frequency-domain features and their respective equations that were considered in this work.

**Time-Domain feature**	**Equation**
Spectral entropy	$H=-\sum_{i}p_{i}\cdot log_{2}(p_{i}),$ with $p_{i}=\frac{P_{i}}{\overset{L-1}{\underset{i=0}{\sum }}P_{i}}$
Mean spectral power	$\bar{P}=\frac{1}{L}\cdot \overset{L}{\underset{i=1}{\sum }}P_{i}$
Maximum spectral power	*max*_*P*_ = max(*P*)
Spectral power variance	$var_{P}=\frac{1}{L}\overset{L}{\underset{i=1}{\sum }}{\left(P_{i}-\bar{P}\right)}^{2}$
Band power	$BP=\overset{f_{2}/\Delta f}{\underset{i=f_{1}/\Delta f}{\sum }}P_{i}$, with $\Delta f=\frac{1}{t_{w}}$
Epileptogenicity index	$Epi_{index}=\frac{P_{\beta }+P_{\gamma }}{P_{\theta }+P_{\alpha }}$

*P is the power spectrum vector, obtained by the DFT, f_1_ the lower band frequency, f_2_ the upper band frequency of the band power, and t_w_ the duration of the sampled window in the time-domain*.

##### Random Forest Classifier

For the classifier itself, the highest possible energy consumption was considered, whereby all 100 trees are used for classification and the tree branches are developed to the maximum depth, which, in this case, was set to 10.

#### Convolutional Neural Network and Recurrent Neural Network Energy Estimation

The same scheme was applied for estimating the AOs for the proposed CNN and RNN models, where each processing layer was considered individually. For the CNN model, all the convolutional layers were estimated according to the standard CNN implementation. Batch normalization parameters were incorporated into the CNN filter parameters. The same applies to the RNN, where the original LSTM implementation was considered. For calculation of hyperbolic tangent and sigmoid activation functions, look-up tables with approximated values were used in both architectures. A more detailed description of the individual layers can be found in the Supplementary Material section.

### Energy Estimation Validation

To validate the feasibility of estimating energy consumption based on the number of MAs, instructions and AOs, the RNN classifier was implemented into an ultra-low-power Apollo 3 Blue microcontroller from Ambiq (Austin, Texas, USA). The energy consumption was measured and compared to the energy calculated.

The energy consumption was measured with the Texas Instrument EnergyTrace™ technology for different numbers of LSTM cells, *N*_*LSTM*_∈{2, …, 20}. The LSTM cells in the model were decreased stepwise from 20 to 2, and the corresponding classification energy consumption was determined for each step.

The validity of the model was proven by analyzing the correlation between measured and calculated classification energy. For this purpose, a linear ordinary least squares regression was performed using the Statsmodels toolbox, which is a Python module (46). The quality of the fit was investigated by evaluating the residuals of the fit with the adjusted R^2^-value (R^2^: coefficient of determination). In addition, a *t*-test was conducted to investigate the significance (significance level: 5%) of the estimated coefficients. Pearson correlation coefficients were calculated using SciPy, a free and open-source python library, as measure of correlation between the two values.

## Results

In this section, the results are presented in two parts. First, the implemented seizure detectors are compared by using several metrics to evaluate and compare their performance. Using these metrics provides valuable insight into the characteristics of these seizure detectors as well as their suitability for closed-loop applications. In the second part, the results of the power consumption estimation for the proposed seizure detection algorithms are presented. These estimations are based on hardware implementation on the Apollo 3 Blue microcontroller.

### Classifier Performance

**Figure 3:**
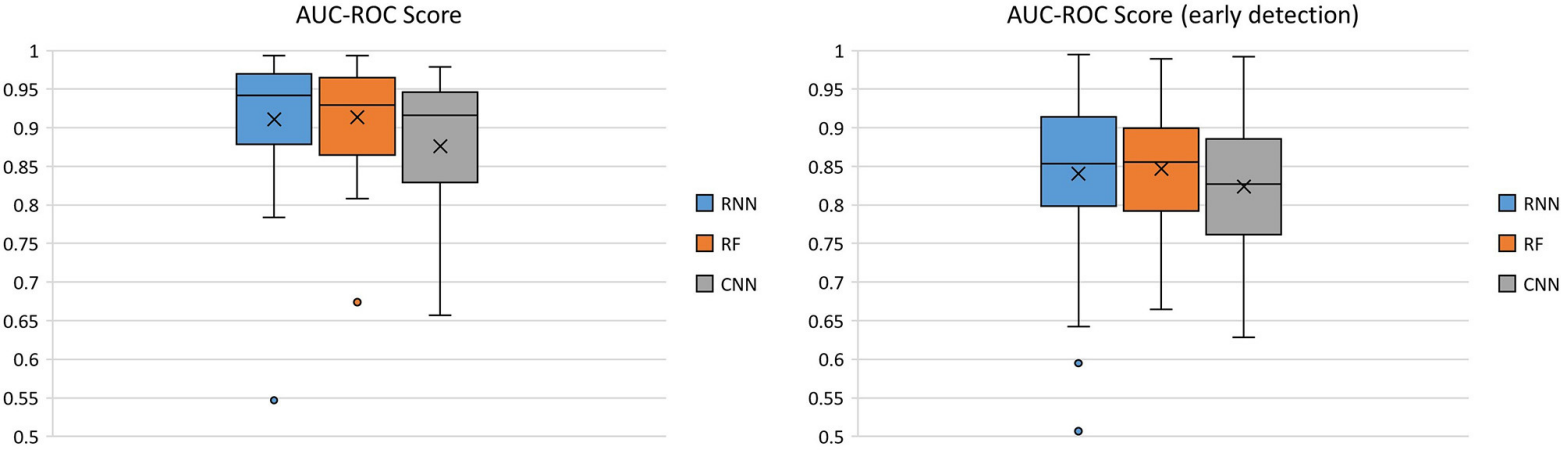
Comparison of the three classifiers across 40 patients using the AUC-ROC score as the performance metric.

Boxplots were selected for visualization of the results because they display the spread of the plotted variable and provide an indication of the variable distribution, such as symmetry and skewness. Moreover, boxplots display the outliers, which help in the understanding how often a classifier fails to perform robustly. The AUC-ROC scores of the three seizure detection algorithms are shown in Figure 3.

For the RNN classifier, two types of input were used and their performance was evaluated. Application of the time-derivative of the data, in place of using the raw data as inputs to the RNN, improved the mean AUC-ROC across all patients ~2.5% for normal seizure detection, and 6.4% for early seizure detection, respectively. For this reason, the results of the time-derivative are presented here.

The median (mean) AUC-ROC score was the highest across all patients for the RNN 0.941 (0.910), compared to that of the RF 0.929 (0.914) and CNN 0.916 (0.876). The average AUC-ROC score for early seizure detection was similar between the RNN 0.853 (0.840) and the RF 0.855 (0.847), followed by CNN 0.827 (0.824).

**Figure 4:**
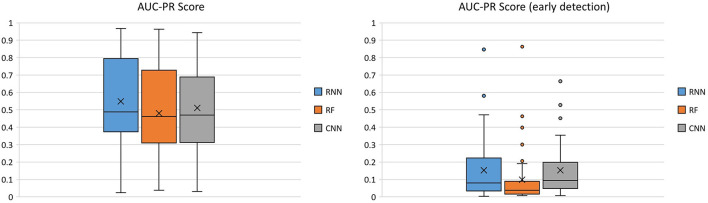
Comparison of the three classifiers across 40 patients using AUC-PR score as the performance metric.

Due to the fact that “ictal” and “interictal” classes are very imbalanced, another useful measure of prediction success is the AUC-PR score. The PR curve shows the trade-off between the precision and recall of different thresholds. The AUC-PR score of the three classifiers across 40 patients is shown in Figure 4.

The RNN had the highest median (mean) AUC-PR score across all patients [0.487 (0.548)], compared to that of the RF 0.462 (0.481), and the CNN 0.470 (0.511). The average AUC-PR score for early seizure detection was similar between the RNN 0.080 (0.153) and CNN 0.093 (0.152), but lower for the RF 0.038 (0.099).

**Figure 5:**
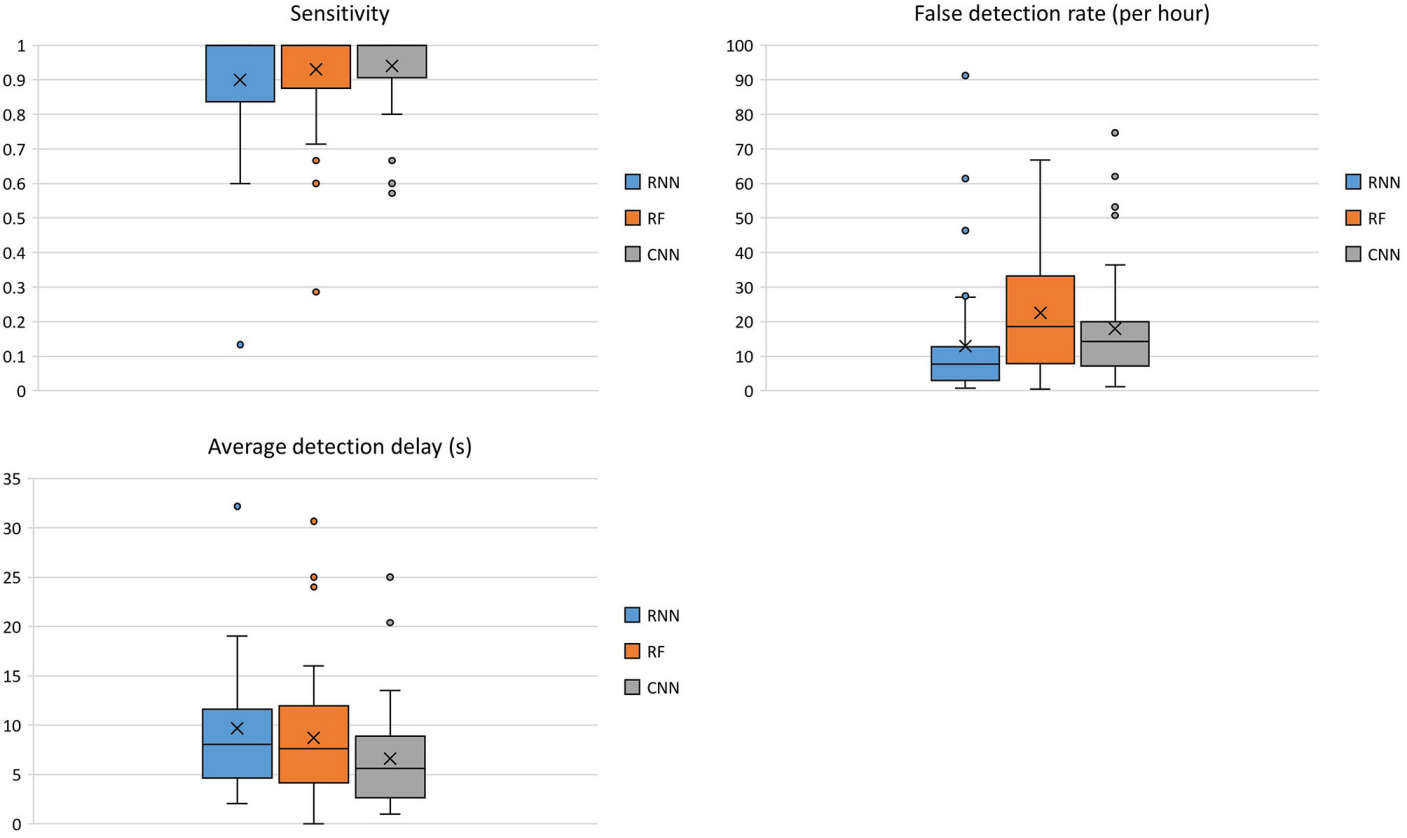
Comparison of the seizure detectors across 40 patients using sensitivity, FDR (per hour), and average detection delay (s) as the performance metrics.

For a more intuitive representation of seizure detector performance, sensitivity (Figure 5), FDR (per hour), and average detection delay (in seconds) for optimized probability thresholds, based on F1-score, were calculated for all seizure detectors across the 40 patients.

Median (mean) sensitivity across all 40 patients were 1.0 (0.90) for the RNN, 1.0 (0.93) for the RF, and 1.0 (0.94) for the CNN. The FDR (per hour) across all patients was 7.77 (12.93) for the RNN, 18.59 (22.49) for RF, and 14.25 (17.89) for CNN. The median (mean) average detection delay (s) across all patients was 8.05 (9.70) for the RNN, 7.65 (8.70) for RF, and 5.60 (6.62) for CNN.

### Energy Estimation

**Figure 6:**
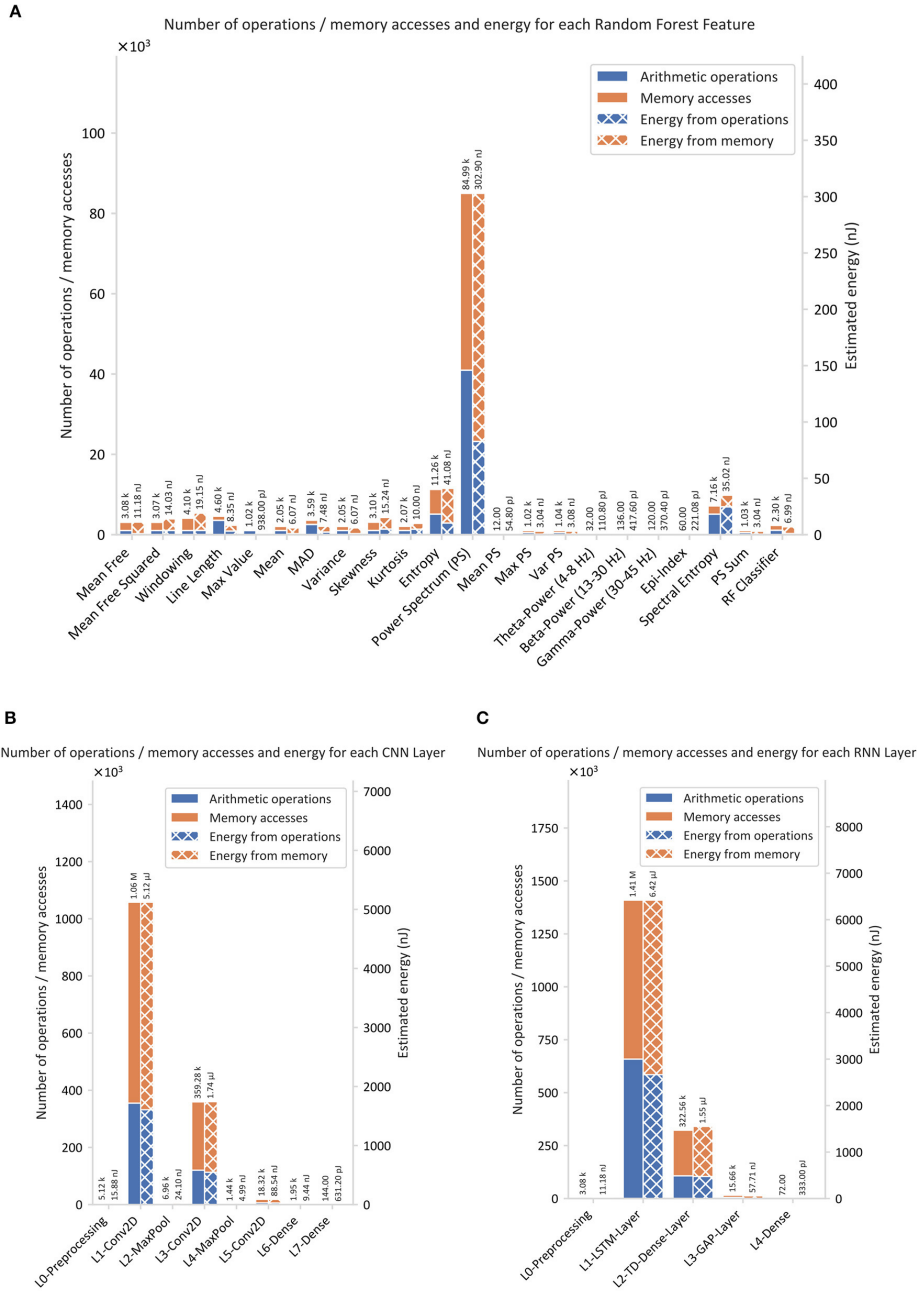
Estimated number of arithmetic operations, memory accesses, and energy using the proposed method: **(A)** RF, **(B)** CNN, and **(C)** RNN.

**Figure 7:**
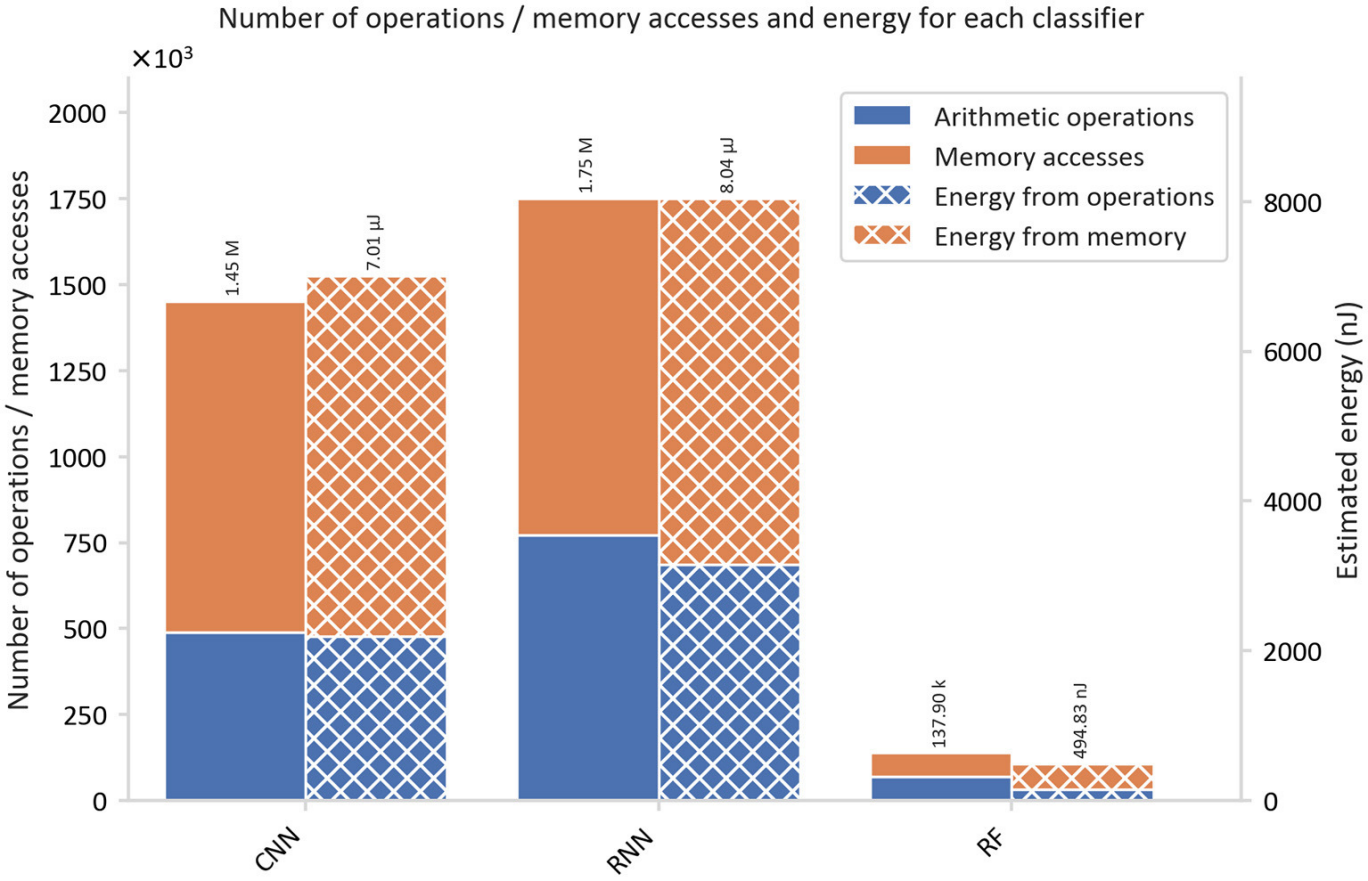
Comparison of the classification energy consumption and the number of operations for the proposed seizure detection classifiers.

The total number of MAs and AOs required for hardware implementation of the proposed seizure detection algorithms is shown in Figures 6, 7. A window size of *t*_*w*_ = 1*s* was chosen, resulting in a number of samples, *N*_*w*_ = *F*_*s*_·*t*_*w*_ = 256, multiplied by the number of channels, *C* = 4.

Figure 6A shows the number of MAs, AOs, and the resulting energy estimation for the calculated time and frequency domain features of EEG as input for the RF classifier. Thereby, it underlines the difference of energy consumption among the different features. The most energy intense operations are required to calculate the power spectrum, the spectral entropy, and the entropy.

Figure 6B shows the number of MAs, AOs, and corresponding energy for every layer of the CNN. It can be observed that the network consumes most of the energy in the convolutional layers. About 69% of the energy in the convolutional layers relates to MAs.

Figure 6C shows that the LSTM layer is by far the most energy intense layer of the RNN. The energy ratio of energy related to MA is about 58% of 6.42 μJ. The Time-Distributed (Dense) layer, however, shows with 69% of 1.55 μJ the same behavior as the convolutional layer in the CNN.

Figure 7 compares the three algorithms and underlines the superiority of the RF classifier over the RNN and the CNN in terms of energy consumption. RNN requires 16.6 times and the CNN 14.5 times more energy than that consumed by the RF.

The total number of AOs required for CNN, RNN, and RF were estimated as denoted in Table 6.

**Table 6:** Estimated energies, number of arithmetic operations, and memory accesses for the CNN, RNN, and RF classifier.

**Classifier**	**Energy (μJ)**			**Number of operations ( ×10** ^**3**^ **)**	
	**Total**	**Operations**	**Memory**	**Arithmetic**	**Memory**
			**accesses**	**operations**	**accesses**
CNN	7.01	2.19	4.81	488	963
RNN	8.04	3.15	4.89	772	978
RF	0.495	0.147	0.348	68.4	69.5

### Energy Estimation Validation

**Figure 8:**
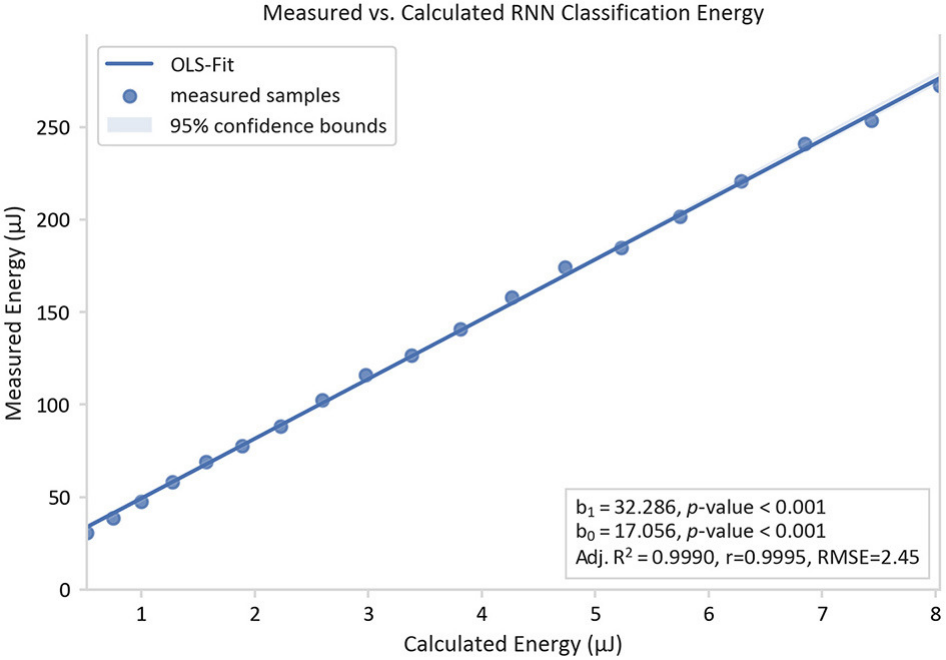
Measured classification energy over the calculated energy and its linear regression curve. The energies are determined by measuring the energy of an RNN implementation on an Apollo 3 Blue ARM Cortex-M4F microcontroller unit under the variation of the number of LSTM cells from 2 to 20.

The energy model was evaluated based on the RNN implementation. The results showed that all the coefficients of the fitted linear regression (as shown in Figure 8) are significant.

A linear trend with a slope b_1_ of 32.29 and an offset b_0_ of 17.06 μJ were observed for the RNN implementation using an Apollo 3 Blue ARM Cortex-M4F microcontroller. The model was proven to be accurate with an adj R^2^ value of 0.9990 and a high correlation coefficient r of 0.9995. As b_1_ serves as a hardware-dependent scaling factor, it follows that the measured energy consumption for this specific hardware setup is over the estimated energy consumption by this factor. Its high value in this case is due to the instruction overhead, which is not part of the model. Furthermore, the offset b_0_ is explained by the hardware-dependent static power consumption in the target system.

An oscillation of the residuals of the linear fit with varying number of LSTM cells can be observed in Figure 8. A possible cause of this phenomenon is the loop-unrolling of the pre-compiled ARM DSP-library. A loop unrolling with a factor of 4 is applied to the vector dot-product function that triggers the running of extra loop-overhead code for vectors longer than a multiple of 4.

## Discussion

In this study, the development of seizure detection algorithms for integration into responsive neurostimulators was addressed. As such a system needs to be implantable into the patient's body, there are considerable restrictions in terms of computational load and energy consumption. Considering these aspects, three seizure detection algorithms were proposed and their performance, as well as the required energy for their implementation in embedded systems, were estimated and compared. Results of the performance comparison showed that the RNN classifier outperforms the other classifiers. Comparison of the required energy using an energy model based on the respective numbers of specific AOs and MAs revealed that the RF classifier is the most efficient seizure detection algorithm, followed by the CNN and RNN. To evaluate the accuracy of the energy estimation of the seizure detection algorithms, the RNN classifier was implemented on an ultra-low-power microcontroller.

The introduced energy estimation model showed good compliance with the energy measurements of the RNN implementation running on an ARM Cortex-M4F-based microcontroller. This notion was validated for the RNN classifier by a significant constant factor in the linear regression fit of the estimated energy with the measured energy over varying numbers of LSTM cells.

A comparison of energy consumption with the existing “RNS device” (Neuropace Inc., Mountain View, CA, USA) is difficult to perform, since detailed information about the energy consumption of the seizure detection algorithms are missing. Only a rough estimation on the basis of battery capacity and longevity for the whole system, including the required energy for stimulation, can be performed. The overall power for a patient profile with low stimulation frequency is ~40 μW. Nevertheless, more detailed information is available for some other prototype devices. For example, a modified ActivaPC device from Medtronic (Minneapolis, Minnesota, USA), which was developed for bi-directional brain machine interfaces by Stanslaski et al. (47), offers 8 MB of RAM. It can operate in a time-domain mode, with a power consumption of 100 μW per channel and a spectral mode with a power consumption of 5 μW per channel. A simple classification stage offers a linear support vector machine with a typical power consumption of 5 μW per channel (47). This is comparable to the power consumption of the proposed models in this study based on RNN (68.1 μW per channel), CNN (60.8 μW estimated per channel), and RF (8.3 μW estimated per channel). An interesting comparison between local feature computation (2.1 μW) and external classification (43 μW) with radio transmission of the data (1,733 μW) for external processing is provided in a study by Verma et al. (48), which clearly favors the use of low-power processing on the edge device over transferring the data to a more powerful external device.

RNN classifiers were used for seizure detection in several recent studies. For example, Abbasi et al. (49) proposed a seizure detection algorithm in which an LSTM architecture with double-layered memory units was applied. They reported a sensitivity of 96.7% on the Bonn University dataset (49). Their proposed network consisted of 100 and 128 LSTM cells in the first and second layers, respectively, which results in a considerably higher computational load and a subsequent higher energy cost compared to the proposed architecture in this study. Similarly, Ahmedt-Aristizabal et al. (50) proposed a seizure detection algorithm based on the RNN-LSTM. They used an LSTM-NN architecture with two subsequent LSTM layers (128 and 64 cells) and obtained an AUC-ROC of 98.52% on the dataset from University of Bonn (50). In another study, Hussein et al. (43) introduced an LSTM architecture where raw EEG data in sequences of 23.6 s were passed on to a recurrent layer with 80 LSTM cells followed by a fully connected layer with 80 cells, a global average pooling layer and a 2-cell classification layer. They reported this methodology as having 100% accuracy, sensitivity, and specificity on the Bonn University dataset (43). As previously mentioned, the model proposed by Hussein et al. influenced the architecture of the RNN model described in the current study, which was modified to contain just one recurrent layer with 20 LSTM cells. This modification then renders the RNN model a better candidate for application in implantable devices. In addition, the use of a much more comprehensive dataset in the current study enables more precise performance evaluation.

Similar to the RNN, CNN and RF have been recently employed for seizure detection (51, 52). For example, Hugle et al. (53) proposed a CNN model for the early detection of seizures from intracranial EEG signals that was designed for implementation on a low-power microcontroller. They reported a median sensitivity level of 0.96, an FDR of 10.1 per hour, and a detection delay of 3.7 s. In comparison, the present study found median sensitivity level of 1, an FDR of 14.25 per hour, and a detection delay of 5.60 s. As there are differences in the signal characteristics of the applied dataset in these two studies, a direct comparison is not possible. In an earlier study from the current research group, Manzouri et al. (54) proposed a seizure detection algorithm based on RF for efficient hardware implementation in implantable devices. The proposed model was similar to that described in the current study, however; 10 features for classification were applied, and a median AUC-ROC score of 0.89 was obtained, compared to 0.93 in the present study. However, because a dataset of intracranial recordings which have a higher signal-to-noise ratio (55), was used in Manzouri et al. (54), a direct comparison of classifier performance with the present study is not possible.

Regarding power consumption, Liu and Richardson (56) implemented and compared the power consumption of a DNN, CNN, and LSTM-based model on the CHB-MIT database. The median (mean) sensitivity of the suggested DNN, CNN, and LSTM models were 0.857 (0.874), 1.00 (0.967), and 1.00 (0.976), respectively, compared to 1.0 (0.899) for RNN and 1.0 (0.940) for CNN in the present study. Although these values are similar between the two studies, they are not directly comparable due to the use of different datasets. By applying a sliding window-based weighted majority voting algorithm, Liu et al. reduced the FDR and reported values of 0.14 (0.169) 1/h for the DNN, 0.084 (0.102) 1/h for the CNN, and 0.063 (0.071) 1/h for the LSTM (56), all of which are lower than those of the current study. Although the deep learning models applied by Liu et al. showed high performance, they exceed the complexity of the seizure detection models proposed in the current study. The CNN model with the best performance-to-energy trade-off proposed by Liu et al. requires 2.4M MAC operations, whereas the proposed CNN model in the current study requires only 472k. The higher complexity results in a high demand for memory, inference time and power consumption.

Since the dataset used in the study is selected based on the geometry of the suggested subgaleal electrodes and is not yet publicly available, it is not possible to perform a one-to-one comparison with other studies that used public datasets such as the CHB-MIT Scalp EEG database (57) or the EPILEPSIAE database (58). Nevertheless, the results of this study allow a direct comparison between three state-of-the-art algorithms for such an electrode setup, which may have similar properties to future implantable devices. Sub-clinical seizures and their impact on seizure detection algorithm performance were not investigated in this study. Sub-clinical seizures are defined as electrographic seizures with rhythmic ictal discharges that evolve in frequency and space, without any subjective or objective alteration in behavior or consciousness (59). Indeed, the development of more robust seizure detectors may be facilitated by including sub-clinical seizures in the performance analysis of seizure detection algorithms. Another aspect to consider during the evaluation of seizure detection algorithms is the strong imbalance between ictal and inter-ictal classes. Evaluation of the proposed models over longer periods of recordings can provide a more realistic representation of the clinical performance of these models in long-term and ultra-long-term recordings.

Regarding energy estimation, the influence of the code compilation process on energy estimation was not investigated. However, incorporating this aspect into the analysis would give an overview of how different compiler settings influence the energy demand of the implemented algorithm.

Different hardware implementations of the seizure detection algorithms could be conceived by designing application-specific integrated circuits. For example, specialized hardware accelerators could be built to reduce the number of required MAs. In this case, the applicability of the model is limited. Besides, adjustment of the model is necessary because some neural network weights can be preserved in the hardware registers. As a result, less MA overhead is needed. However, the model helps to identify the aspects which are key to the design of ASICs. Moreover, it aids in selecting the right accelerator for the chosen algorithm.

How the energy consumption of the proposed classifiers can be further optimized will be investigated in future studies. One possibility for this is the application of pruning (22) or quantization techniques. The latter allows for the use of single-instruction multiple-data instructions to perform AOs on multiple data, instead of only two operands in a typical microprocessor setup. Horowitz (45) outlined how this method saves energy by decreasing the ratio of instruction overhead for the same number of AOs. He also suggested using integer calculations with small bit-widths to reduce energy consumption. The intensive use of single-instruction multiple-data for neural network applications was proposed by Lai et al. (60) by introducing the CMSIS-NN library from ARM Ltd. (Cambridge, England, UK). A more modern approach is the application of DNN-Accelerators. For microcontroller systems, one possibility was introduced by ARM with the Ethos neural processing unit. Among other things, they improved the memory access of network parameters, which showed significant improvement in inference, speed, and power consumption (61).

The proposed methodology for energy estimation in the current study can be used to verify both the suitability and applicability of the developed seizure detection models for implantable devices, and provides a reliable estimation. Furthermore, the three proposed models in this study are all candidates for utilization in implantable devices and can be selected based on the specific requirements, limitations, and application of the implantable device.

## Data Availability Statement

The original contributions presented in the study are included in the article/Supplementary Material. Further inquiries can be directed to the corresponding author.

## Ethics Statement

The studies involving human participants were reviewed and approved by Ethics commission of the Albert-Ludwigs-Universität Freiburg, Germany. Written informed consent to participate in this study was provided by the participant or the participant's legal guardian/next of kin. Informed consent of the patient covered the in-house use of EEG recordings.

## Author Contributions

FM and MZ: writing, conceptualization, algorithm development, and implementation. SS: algorithm development and implementation. MD: conceptualization, data curation, and supervision. RM and LC: conceptualization and supervision. PW and AS-B: conceptualization, supervision, and funding acquisition. All authors contributed to the article and approved the submitted version.

## Conflict of Interest

The authors declare that the research was conducted in the absence of any commercial or financial relationships that could be construed as a potential conflict of interest.

## Publisher's Note

All claims expressed in this article are solely those of the authors and do not necessarily represent those of their affiliated organizations, or those of the publisher, the editors and the reviewers. Any product that may be evaluated in this article, or claim that may be made by its manufacturer, is not guaranteed or endorsed by the publisher.
