# Supplementary material for: A Comparison of Energy-Efficient Seizure Detectors for Implantable Neurostimulation Devices
Source: Front Neurol. 2022 Mar 4;12:703797. doi: 10.3389/fneur.2021.703797 (PMC8934428; doi:10.3389/fneur.2021.703797)
Supplement: Supplementary file 1 [file Data_Sheet_1.pdf]

Materials and Methods Supplementary

In this document, the energy estimation methodology is described in more detail. First, a description of the energy estimation for the set of features used as input for the random forest (RF) classifier and the classifier itself is provided. Next, estimation of the required energy for the proposed convolutional neural network (CNN) model is described. Finally, energy estimation for the recurrent neural network (RNN) classifier is explained. The energy estimation is given in terms of the number of arithmetic operations and memory accesses.

1 Time-domain Features for Random Forest Classifier

For a subset of features including mean absolute deviation (MAD), variance, kurtosis, and skewness, which require a common set of operations, they are computed only once in advance and stored into the memory. Via these operations, zero-mean values of the signal and their square values are calculated. For calculating the zero-mean values, the N inputs and the mean value are loaded, processed, and restored into the memory: $load_{mean-free} = N + 1$, $add_{mean-free} = \text{N}$, and $store_{mean-free} = N$.

The same procedure is performed for the calculation of their square values with $load_{(mean-free)^2} = N$, $mult_{(mean-free)^2} = \text{N}$, and $store_{(mean-free)^2} = N$.

1.1 Maximum

To calculate the maximum value each element is compared to the previously found maximum. Therefore, N loads are required, where N is the number of elements in the array. As each element is compared to the previously selected maximum and as the first value in the input vector is set as initial maximum, N-1 comparisons are executed. The resulting maximum value is stored once into the memory.

1.2 Mean

The arithmetic mean value is calculated as $\overline{x} = \frac{1}{N} \cdot \sum_{i=1}^{N} x_i$. Thus, it consists of N loads, where N is the number of input data, N-1 add operations, one multiplication, and one store operation.

1.3 Mean Absolute Deviation

The estimation of the MAD is based on the following formula: $MAD = \frac{1}{N}\sum_{i=1}^{N}|x_i - \bar{x}|$. For its calculation, N+1 values are loaded: N zero-mean values and a known positive value. The latter is the second parameter of N compare instructions, used to estimate the sign of the zero-mean values.

Due to high pass filtering, the EEG signal has a zero mean value (no offset). Assuming a Gaussian distribution for the EEG data, the probability of a negative signal value is equal to the probability of a positive one, therefore the probability that an operation must be performed to make the zero-mean value positive is $P_{sub} = 0.5$. This assumption results in $N - 1$ additions to sum up the zero-mean

DOI: 10.3389/fneur.2021.703797

values and $P_{sub} \cdot N$ additions to obtain the sum of absolute values. It follows one multiplication and a store operation.

1.4 Variance

The empirical variance is calculated as $var = \frac{1}{N}\sum_{i=1}^{N}(x_i - \bar{x})^2$. This requires N loads of the squared zero-mean values, N-1 additions, one multiplication and a store operation.

1.5 Skewness

The sample skewness is computed as the Fisher-Pearson coefficient of skewness, as follows: $g_1 = \frac{m_3}{m_2^{3/2}}$ where m_k is the biased sample kth central moment, and $\bar{x}$ is the sample mean: $m_k = \frac{1}{N}\sum_{i=1}^{N}(x_i - \bar{x})^k$. To calculate the skewness, first, the zero-mean values, the squared zero-mean values, and the variance (m_2) are loaded, resulting in $2N + 1$ loads. Next, to calculate the numerator of g_1 (m_3), zero-mean and squared zero-mean values are used to calculate the sum through $N - 1$ multiply-accumulate (MAC) operations and one multiplication. Then, the calculated sum is multiplied by $\frac{1}{\mathrm{N}}$. Subsequently to calculate the denominator of g_1 ($m_2^{3/2}$) the variance is multiplied with the square root of itself. Finally, a division is required for the calculated numerator and denominator. Then the result is saved using a store operation.

1.6 Kurtosis

For the Kurtosis, the calculation procedure used for skewness can be followed with minor changes. The kurtosis is defined as follows: $g_2 = \frac{m_4}{m_2^2} - 3$ where m_4 and m_2 are the fourth and second sample moment about the mean, respectively, and are calculated using the equation introduced in the previous section. In this case, only the squared zero-mean values must be loaded, and no standard deviation is required. An overview of the required operation follows: $load_{kurt} = N$+1, $MAC_{kurt} = N - 1$, $mult_{kurt} = 3$, $div_{kurt} = 1$,and $store_{kurt} = 1$.

1.7 Line Length

Line length for a single window is defined as $LL = \frac{1}{K} \cdot \sum_{k=2}^{N}|x_k - x_{k-1}|$ (1), where the constant normalization factor can be dropped to save computations, resulting in $LL = \sum_{k=2}^{N}|x_k - x_{k-1}|$. For the calculation, N input values are loaded from memory and each value is subtracted from the previous one. The sign of the results of the subtractions is proved with the compare operation and, in case of negative value, its inversion is required. This allows to obtain the sum of absolute values, as seen for the MAD calculation. The $N - 1$ subtractions and $\frac{N}{2}$ addition operations required for the change of sign of the negative subtraction values are added to the $N - 1$ add operations to calculate the sum. One store operation is required for the final result.

1.8 Entropy

DOI: 10.3389/fneur.2021.703797

The information entropy is defined as: $H = -\sum_i p_i \cdot log_2(p_i)$, where p_i is the probability of occurrence of a symbol i in a set of symbols (2). The probabilities of occurrence of data in the input vector is estimated using a histogram. The input values are sorted to the belonging bin by scaling them by a factor $\frac{1}{2^{bs}}$, where bs denotes the related bit shift in integer arithmetic. Then, the values are converted to integer to address and increment the relevant count variable. Depending on the range of the values A_{max}, and the number of bins N_{bins}, bs is retrieved as $bs = \frac{ceil(log_2(A_{max}))}{log_2(N_{bins})}$. The result of the multiplication $p_i \cdot log_2(p_i)$ is gained using the counts as input for a lookup table of width N, as N is the maximum number of occurrences a count can have. Finally, the loaded results for each count variable are summed. The complete set of operations required for this feature are $load_{entropy} = N + 2N_{bins}$, $mult_{entropy} = N$, $float2int_{entropy} = N$, $add_{entropy} = N + N_{bins} - 1$, and $store_{entropy} = N + 1$.

2 Frequency-domain Features for Random Forest classifier

The calculation of the frequency-domain features is based on the power spectrum that is obtained using the discrete Fourier transform (DFT). To minimize spectral leakage, a window function was applied before performing the DFT. The application of a window function to the input data consists of the multiplication of every data point with the values of the window function stored in memory. Thus, the input data and the window function need to be loaded, multiplied, and stored again. This results in 2N loads, N multiplications, and N store operations. For implementation, the Cooley-Tukey Radix-2 FFT algorithm (3) was chosen. According to (4) the number of complex multiplications and additions in the FFT can be computed as $\mathrm{complexMult_{FFT}} = \frac{\mathrm{M}}{2} \cdot \log_2\left(\frac{\mathrm{M}}{2}\right)$ and $\mathrm{complexAdd_{FFT}} = M \cdot \log_2(\mathrm{M})$, where M is the number of input samples. The multiplication of two complex numbers consists of 4 real multiplications and 2 real additions, such that $\mathrm{mult_{FFT}} = 4 \cdot \mathrm{complexMult_{FFT}}$. The complex addition corresponds to 2 real additions, that together with the additions from the complex multiplication, result in $\mathrm{add_{FFT}} = 2 \cdot \mathrm{complexAdd_{FFT}} + 2 \cdot \mathrm{complexMult_{FFT}}$.

The number of load and store operations depends on the number of stages that constitute the FFT. In every stage, the twiddle-factors for the FFT and the input data of the corresponding FFT-stage need to be loaded from memory. Given that the number of stages in the FFT is $\mathrm{stages_{FFT}} = \log_2(\mathrm{N})$, the total loaded values are $load_{FFT} = stages_{FFT} \cdot 3\mathrm{M}$, and the total stores are $store_{FFT} = stages_{FFT} \cdot 2\mathrm{M}$.

For calculating the power spectrum, the arithmetic and store operations required for the FFT must be counted. The Fourier transformed signals must be squared, summed up and the M results stored, resulting in $mult_{PS} = M \cdot 2$, $add_{PS} = M$, $load_{PS} = 2 \cdot M$, $store_{PS} = M$.

2.1 Spectral Entropy

The spectral entropy probabilities of the power of each frequency bin are calculated by normalizing the signal power P_i of frequency bin i. This is done by dividing it by the overall signal power $\sum_{i=0}^{N-1} P_i$: $p_i = \frac{P_i}{\sum_{i=0}^{N-1} P_i}$. Different from the entropy calculation in the time domain, where amplitudes are counted, no aggregation of values is performed for the spectral entropy. Therefore, a direct data retrieval would require a big lookup table, which is not appropriate for this application. This issue is resolved by using a smaller lookup table with linear interpolation between the points. The operations required for the

table look up are $load_{LUT} = 2$, $float2int_{LUT} = 1$, $int2float_{LUT} = 1$, $mult_{LUT} = 3$, $add_{LUT} = 3$, $store_{LUT} = 1$.

As for the entropy a lookup table containing the multiplication $p_i \cdot log_2(p_i)$ was used, the spectral power values are summed up and each spectral power bin is normalized with the signal power. The results are accumulated until all spectral bins are considered. The resulting operations are $load_{SE} = \frac{N}{2} + \frac{N}{2}$, $div_{SE} = \frac{N}{2}$, $LUT - ops_{SE} = \frac{N}{2}$, $add_{SE} = \frac{N}{2} + \frac{N}{2} - 2$, and $store_{SE} = 2$.

2.2 Mean Spectral Power

The mean spectral power is the signal power divided by the fixed number of frequency bins. As the total spectral power is calculated for the spectral entropy, the required operations are $load_{mean-SP} = 1$, $mult_{mean-SP} = 1$, and $store_{mean-SP} = 1$.

2.3 Maximum Spectral Power

The maximum spectral power is found with the same algorithm used for the maximum value in the time-domain features. However, the number of values to be loaded decreases to $\frac{N}{2}$, such that $load_{max-SP} = \frac{N}{2}$, $comp_{max-SP} = \frac{N}{2} - 1$, $store_{max-SP} = 1$.

2.4 Spectral Power Variance

The spectral power variance is defined with the following equation: $var_{SP} = \frac{2}{N}\sum_{i=1}^{N/2}(P_i - \bar{P})^2$. In this case $(P_i - \bar{P})^2$ needs to be calculated. The calculations required for this feature are obtained by combining the operation to calculate $(P_i - \bar{P})^2$ with the variance calculation in the time domain. As a result, $load_{var-SP} = \frac{N}{2} + 1$, $add_{var-SP} = \frac{N}{2}$, $MAC_{var-SP} = \frac{N}{2}$, $mult_{var-SP} = 1$, and $store_{var-SP} =1$ is the computational load.

2.5 Band Power

The spectral power for each band is calculated by summing up the stored power spectrum values of the correspondent frequency range. Hence, the number of bins to sum depend on the bandwidth B of the feature and the frequency resolution $\Delta f = \frac{1}{t_w}$ of the FFT, where t_w is the length of the window in the time domain. The number of loads is then calculated as $load_{bandpower} = \frac{B}{\Delta f}$, the additions are $add_{bandpower} = \frac{B}{\Delta f} - 1$, and storing the result leads to $store_{bandpower} = 1$.

The band power is calculated in the following frequency ranges: theta band (4-8 Hz) (5), alpha band (8-13 Hz) (6), beta band (13-30 Hz), and gamma band (30-45 Hz).

2.6 Epileptogenicity Index

The epileptogenicity index is defined as the ratio of the power in higher frequency bands (beta + gamma) to the lower frequency bands (theta + alpha): $Epi_{index} = \frac{P_\beta + P_\gamma}{P_\theta + P_\alpha}$. For its calculation, the

DOI: 10.3389/fneur.2021.703797

previously stored power values are loaded. The required load, arithmetic, and store operations are as follows: $load_{epi} = 3$, $add_{epi} = \frac{9\,Hz}{\Delta f} + 1$, $div_{epi} = 1$, and $store_{epi} = 1$.

3 Random Forest Classifier

To estimate of the required energy for the RF classifier, the highest possible energy consumption was assumed. Consequently, an ensemble of 100 decision trees, which are grown to their maximum allowed depth, was considered for the estimation. This model results in 1000 compare operations, and 1100 memory accesses.

4 Convolutional Neural Network Energy Estimation

In the following, the energy estimation for each layer of the proposed CNN architecture is discussed.

4.1 Preprocessing

The scaling of the input data by the standard deviation can be combined with the constant factor 0.2 in the equation $\hat{x}(t) = tanh(0.2 \cdot x(t))$, resulting in a single parameter multiplication. The linear part in the piecewise linear approximation of the tanh function is merged into this operation. For the upper and lower bounds of the tanh function, a comparison with the threshold values is required. All input values and the thresholds must be loaded to be processed, resulting in $load_{preprocess} = N \cdot C + 1$. The required number of multiplications and comparisons is $mult_{preprocess} = N \cdot C$, $comp_{preprocess} = 2 \cdot N \cdot C$, and to store the result, the following amount of store operations is needed: $store_{preprocess} = N \cdot C$.

4.2 Convolutional Layers

The output of a 2-D convolutional layer consists of a 2-D feature map for each of the filters $F_i \in \mathbb{R}^{x \times y}$ for $i \in \{1, \ldots, N_{filt}\}$, where x is the filter length in the first dimension, which corresponds to the number of electrodes in the input layer, and y is the filter length in the second dimension, which corresponds to the time axis in the input layer. N_{filt} represents the number of filters in the convolutional layer. A single feature map is produced by striding a filter over the input data array and performing the filter operation in each position. A filter operation consists of the following steps: 1- application of a dot product, which includes multiply and add operations, between the filter weights and the input data that is covered by the filter, and 2- adding the bias value, which is learned during the training phase for each filter, to the dot product. It follows that every filter operation can be reduced to so many MAC-operations as filter elements. For every filter operation, an output of the resulting feature map O_i is generated. $O_i \in \mathbb{R}^{w \times h}$ depend on layer parameters, such as kernel stride and the type of padding that is used. For O_i, the number of MAC-operations for a 2-D convolutional layer is calculated irrespectively of the layer parameters as $MAC_{conv2D} = x \cdot y \cdot w \cdot h \cdot N_{filt}$. To add the bias the following number of additions are required: $add_{conv2D} = w \cdot h \cdot N_{filt}$. Assuming that in a sequential computation logic for each MAC-operation the data and the filter need to be loaded, the number of load operations for the 2-D convolutional layer is estimated as $load_{conv2D} = 2 \cdot MAC_{conv2D} + add_{conv2D}$. The result of each filter operation is stored into memory, thus leading to

DOI: 10.3389/fneur.2021.703797

$store_{conv2D} = w \cdot h \cdot N_{filt}$. As the activation function can be applied directly after calculating the filter output and before storing it to memory, no additional memory access overhead is needed. However, an arithmetic overhead in the form of a comparison is needed for the rectified linear unit (ReLU) activation function to prove if the output is bigger than 0:

$comp_{ReLU} = w \cdot h \cdot N_{filt}$.

4.3 Max-Pooling Layers

Max-pooling performs a max operation by striding a window over the input, operating on a subset of the input data for every stride. The max-pooling operation corresponds to the max operation previously introduced in the feature calculation section. Hence, the same number of arithmetic and memory operations applies for each stride. The multiplication of the number of operations required to calculate the maximum with the number of outputs, as explained for the convolutional layer, gives the total number of operations in the max-pooling Layer.

4.4 Batch Normalization

Because for CNNs, the batch normalization parameters are learned for each feature map, instead of each neuron activation, they can be incorporated into the filter parameters (7). This eliminates the need for batch normalization transforms on the convolutional layers.

4.5 Dense Layer

Dense layer is a synonym for a fully-connected neural network layer, in which every individual neuron of the layer is connected to the previous layer via a weight w_{ij}, where $i \in \{1, \dots, N_{in}\}$ corresponds to the input number with N_{in} total inputs and $j \in \{1, \dots, M_{neurons}\}$ to the neuron number with $M_{neurons}$ total neurons. The network activations $\overrightarrow{net}$ of each neuron in the layer can be calculated using matrix arithmetic with the weight matrix W being

$$W = \begin{pmatrix} w_{11} & \cdots & w_{1M} \\ \vdots & \ddots & \vdots \\ w_{N1} & \cdots & w_{NM} \end{pmatrix},$$

with the input vector $\vec{v} \in \mathbb{R}^N$, and the bias vector $\vec{b} \in \mathbb{R}^M$ as

$$\overrightarrow{net} = \vec{v}^T \cdot W + \vec{b}.$$

As a result of the vector-matrix multiplication, the number of MAC-operations in a dense layer is $MAC_{Dense} = M_{neurons} \cdot N_{in}$. In addition, the following number of additions is required for adding the bias: $add_{Dense} = N_{in}$. Next, the memory accesses are calculated similar to the convolutional layer, considering that the input data, weight, and bias parameters need to be loaded from the memory

$$load_{Dense} = 2 \cdot M_{neurons} \cdot N_{in} + N_{in}.$$

Furthermore, the network output needs to be stored

$$store_{Dense} = M_{neurons}.$$

DOI: 10.3389/fneur.2021.703797

The selected activation function (ReLU, or sigmoid in the proposed model) can be applied before storing the result into memory. The respective operations and memory accesses must be added to the calculations above. A possible implementation of the sigmoid function is shown in 5.2.

5 Recurrent Neural Network Energy Estimation

In this section, given the model layers, the number of arithmetic operations and memory accesses for the RNN model are calculated. For these calculations, 32-Bit floating-point operations are assumed.

5.1 Preprocessing

The input data is preprocessed by calculating the time derivative for each channel and is restored into memory.

5.2 Long Short-term Memory Layer

For calculation of the total number of operations in a long short-term memory (LSTM) layer, the recursive processing of the input is considered. As a result, the operations of an LSTM cell must be calculated for each of the N total time steps as follows: $\mathrm{N_{LSTM-Cells}} \cdot \mathrm{N}_{ts}$ where $\mathrm{N_{LSTM-Cells}}$ is the number of LSTM cells in the LSTM layer and N_{ts} is the total number of time steps, which is equal to the input window length.

To calculate the number of operations in a single cell the following equations based on (8) and (9) are considered, which calculate the internal state of a single LSTM cell at a single time step, where c_t is the internal state value at time step t and h_t the output of the LSTM cell:

$$c_t = c_{t-1} \cdot \sigma\left(net_{f_t}\right) + \sigma(net_{i_t}) \cdot tanh(net_{c_t})$$

$$h_t = tanh\ (c_t) \cdot \sigma(net_{o_t})$$

The network activations net of the gates f, i, o, and the kernel c are calculated with a fully-connected layer, the input of which are multiplied by a weight vector for each LSTM cell, or a weight matrix for all LSTM cells. The length of the weight vector for each activation is $\mathrm{L_w} = \mathrm{N_{feat}} + \mathrm{N_{LSTM-Cells}} + 1$, where $\mathrm{N_{feat}}$ is the number of input features which is equal to the number of EEG-channels, and 1 accounts for the bias weight. The activation functions σ and $tanh$ are implemented using a lookup table. To reduce computational costs, a lookup table was used without interpolation between the points. By using a lookup-table of 8192 points, the maximum absolute error of the sigmoid functions is 4.84×10^{-4}, and the one of the tanh function is 9.62×10^{-4}.

Additionally, it was considered that the internal state c_{t-1} of the LSTM-cells at $\mathrm{t} = 0$ is 0, hence the equation of c_0 reduces to $c_t = \sigma(net_{i_t}) \cdot tanh(net_{c_t})$. Furthermore, as also $\mathrm{h_{t=-1}} = 0$, the calculation of all activations net reduces to the feature weights and the bias at the first time step. The memory accesses to retrieve the network parameters, as well as the accesses to store the results are considered in the calculation.

DOI: 10.3389/fneur.2021.703797

5.2.1 Time-Distributed Dense Layer

The same considerations as for the dense layer of a CNN are made here with two exceptions. The first one is that all results need to be multiplied by N_{ts}, as the dense layer is calculated for each time step. The second is that the activation function is linear, so no additional operation is required as input and output are equal

5.3 Global Average Pooling Layer

The global average pooling layer averages the outputs of the time-distributed dense layer over the time axis t. In this layer, the outputs of the previous layer are accumulated directly after their calculation, thus avoiding store operations for all time steps. Thereafter, they are averaged by dividing them with N_{ts}.

5.4 Output Layer

The output layer consists of a single dense cell with a sigmoid activation function and can be calculated similar to the CNN with $\mathrm{N}_{Dense} = 1$ and with the operations needed for lookup-table-access.

References

1. Esteller R, Echauz J, Tcheng T, Litt B, Pless B. Line length: An efficient feature for seizure onset detection. *Annual International Conference of the IEEE Engineering in Medicine and Biology-Proceedings* (2001) 2:1707–1710. doi:10.1109/IEMBS.2001.1020545

2. Shannon CE. A Mathematical Theory of Communication. *Bell System Technical Journal* (1948) 27:379–423. doi:10.1002/j.1538-7305.1948.tb01338.x

3. Cooley JW, Tukey JW. An Algorithm for the Machine Calculation of Complex Fourier Series. *Mathematics of Computation* (1965) 19:297. doi:10.2307/2003354

4. Kammeyer K-D, Kroschel K. “Die schnelle Fourier-Transformation (FFT),” in *Digitale Signalverarbeitung* (Wiesbaden: Vieweg+Teubner Verlag), 253–274. doi:10.1007/978-3-8348-8627-9

5. Klimesch W. EEG alpha and theta oscillations reflect cognitive and memory performance: a review and analysis. *Brain Research Reviews* (1999) 29:169–195. doi:10.1016/S0165-0173(98)00056-3

6. Noachtar S, Binnie C, Ebersole J, Mauguière F, Sakamoto A, Westmoreland B. *A glossary of terms most commonly used by clinical electroencephalographers and proposal for the report form for the EEG findings. The International Federation of Clinical Neurophysiology.* (1999). Available at: http://www.ncbi.nlm.nih.gov/pubmed/10590974 [Accessed February 15, 2019]

7. Ioffe S, Szegedy C. Batch Normalization: Accelerating Deep Network Training by Reducing Internal Covariate Shift. PMLR (2015).

8. Hochreiter S, Schmidhuber J. Long Short-Term Memory. *Neural Computation* (1997) 9:1735–1780. doi:10.1162/neco.1997.9.8.1735

DOI: 10.3389/fneur.2021.703797

9. Gers FA, Schmidhuber J, Cummins F. Learning to forget: Continual prediction with LSTM. *Neural Computation* (2000) 12:2451–2471. doi:10.1162/089976600300015015

DOI: 10.3389/fneur.2021.703797
